# Thyroid Cancer and Psoriasis: A Nested Case–Control Study

**DOI:** 10.3390/diagnostics12102297

**Published:** 2022-09-23

**Authors:** So Young Kim, Dae Myoung Yoo, Juyong Chung, Hyo Geun Choi

**Affiliations:** 1 Bundang CHA Medical Center, Department of Otorhinolaryngology-Head and Neck Surgery, CHA University, Seongnam 13488, Korea; 2 Hallym Data Science Laboratory, Hallym University College of Medicine, Anyang 14066, Korea; 3 Department of Otorhinolaryngology-Head and Neck Surgery, Wonkwang University School of Medicine, Iksan 54538, Korea; 4 Department of Otorhinolaryngology-Head and Neck Surgery, Hallym University College of Medicine, Anyang 14068, Korea

**Keywords:** thyroid cancer, psoriasis, risk factors, cohort studies

## Abstract

Previous researchers have suggested an elevated risk of thyroid cancer (TC) in patients with psoriasis with mixed results. The current study evaluated the relationship of psoriasis with the risk of TC in an adult population. The data from the Korean National Health Insurance Service—Health Screening Cohort ≥ 40 years old were analyzed. In total, 6822 patients with TC were equalized with 27,288 control participants using overlap weighting adjustment based on the propensity score. The history of psoriasis was compared between the TC and control groups using multivariable logistic regression analysis. Secondary analyses were conducted according to age, sex, income, region of residence, systolic blood pressure, diastolic blood pressure, fasting blood glucose, total cholesterol, obesity, smoking, alcohol consumption, the Charlson Comorbidity Index scores, hypothyroidism, goiter, thyrotoxicosis, and thyroiditis. The history of psoriasis was not different in patients with TC (overlap-weighted odds ratio (OR) = 1.02, 95% confidence intervals (CI) = 0.85–1.22). The <55-year-old group showed a high rate of TC associated with psoriasis (overlap-weighted OR = 1.69, 95% CI = 1.22–2.36, *p* = 0.002). The population without hypothyroidism demonstrated an increased rate of TC related to psoriasis (overlap-weighted OR = 1.29, 95% CI = 1.06–1.57, *p* = 0.012). The patients with hypothyroidism showed a low rate of TC for psoriasis (overlap-weighted OR = 0.59, 95% CI = 0.37–0.96, *p* = 0.034). None of the other subgroups showed an association between psoriasis and TC. Psoriasis was not related to the risk of TC in the overall adult population. Young adults and populations without hypothyroidism indicated an elevated rate of TC for psoriasis.

## 1. Introduction

Thyroid cancer (TC) is the most common endocrine cancer, with increasing incidence [1,2,3]. Thus, many researchers and clinicians have investigated the causes and clinical courses of TC [3,4,5]. Genetic factors, iodine intake, and thyroid disorders have been reported to elevate the risk of TC [5]. Moreover, lifestyle factors, such as obesity and environmental pollutant exposure, have been described as contributors to TC [5]. These risk factors for TC were suggested to induce immune modulation and inflammation, which can be protumorigenic in TC [6]. Therefore, the risk of TC has been investigated in chronic diseases with immune or inflammatory dysfunction, such as diabetes, obesity, and psoriasis [7,8,9].

Psoriasis is a common skin disease with an approximately 2–4% prevalence [10]. The scales originate from premature keratinocytes and parakeratosis in the highly proliferative epidermis [10]. This hyperproliferation and related inflammation of the skin is induced by multiple pathophysiologic causes, including immune dysregulation and genetic factors [10]. In addition, lifestyle factors of obesity, smoking, and alcohol consumption have been supposed to increase the risk of psoriasis [11,12,13]. Due to the pathology involving immune dysfunction, chronic inflammation, proliferative disorder, and the risks associated with lifestyle factors, several researchers proposed a high risk of cancers in patients with psoriasis [9,14,15,16]. For TC, the risk associated with psoriasis has been inconsistent [9,16]. As both psoriasis and TC are linked with chronic diseases and lifestyle factors, these factors should be evenly distributed between the study and control groups, and multivariable analyses are warranted to include these variables.

To fill this gap in knowledge on the influence of psoriasis on TC, we investigated the association of psoriasis with TC. To attenuate the potential confounding effects of lifestyle factors and chronic diseases, these factors were considered by overlap weighting adjustment and adjusted models including these variables. Furthermore, secondary analyses were extensively conducted to identify a certain group associated with psoriasis and TC.

## 2. Materials and Methods

The Korean National Health Insurance Service—Health Screening Cohort data were analyzed [17]. The ethics committee of Hallym University (IRB No: 2019-10-023) permitted the current research.

**Figure 1:**
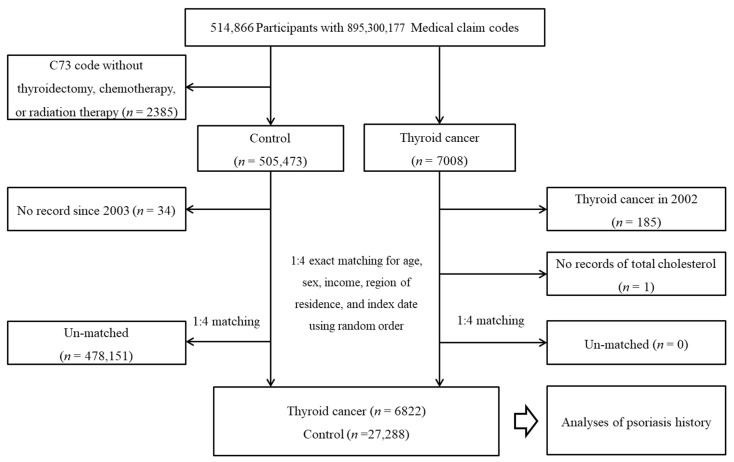
A schematic illustration of the participant selection process used in the present study. Of a total of 514,866 participants, 6822 thyroid cancer participants and 27,288 control participants were matched in a 1:4 ratio according to age, sex, income, and region of residence.

Among a total of 514,866 participants from 2002 through 2019, TC participants were enrolled (n = 7008). The TC participants who were diagnosed in 2002 were excluded. The participants who were diagnosed with TC without thyroidectomy, chemotherapy, or radiation therapy were excluded. The participants who died before 2003 or had no records since 2003 were excluded. The TC participants were randomly matched for age, sex, income, and region of residence with the control participants. As a result, 6822 TC participants and 27,288 control participants were selected (Figure 1).

TC was classified based on the ICD-10 codes (C73). In addition, those patients with TC who underwent thyroidectomy (claim codes: P4551, P4552, P4553, P4554, and P4561), chemotherapy (claim codes: J0041, CX454, KK151-KK156, and KK158), or radiation therapy (claim codes: HD010-HD023, HD031-HD033, HD041, HD051-HD056, HD058, HD059, HD061, HD071-HD073, HD080-HD086, HD088, HD089, HD091-HD093, HD110-HD115, HD121-HD212, HD410-HD416, HD418-HD420, and HD441) were included.

Psoriasis was classified based on ≥2 clinical visits with a diagnosis of psoriasis (ICD-10: L40).

Age groups, levels of income, and regions of residence were classified [18]. Histories of smoking, alcohol consumption, and obesity were classified [18].

The total cholesterol (mg/dL), systolic blood pressure (SBP, mmHg), diastolic blood pressure (DBP, mmHg), and fasting blood glucose (mg/dL) were checked.

The Charlson Comorbidity Index (CCI) score was calculated [19]. TC was not included in the CCI score.

Histories of hypothyroidism (ICD-10 codes: E02 and E03), goiter (ICD-10 codes: E04), thyrotoxicosis (ICD-10 codes: E05), and thyroiditis (ICD-10 codes: E06) were classified based on ≥2 clinical visits.

The propensity score (PS) overlap weighting was conducted. PS was estimated by multivariable logistic regression with all the covariates. PS was applied in which the TC participants were weighted by the probability of PS, and the control participants were weighted by the probability of 1-PS [20,21,22]. The standardized difference after and before weighting was used to compare the differences in general characteristics between the TC and control groups (Table 1).

To analyze the overlap-weighted odds ratios (ORs) of psoriasis for TC, the propensity score overlap-weighted multivariable logistic regression analysis was used. In these analyses, the crude (unadjusted) and overlap-weighted models (age, sex, income, region of residence, SBP, DBP, fasting blood glucose, total cholesterol, obesity, smoking, alcohol consumption, CCI scores, hypothyroidism, goiter, thyrotoxicosis, and thyroiditis) were used (Table 2). The 95% confidence interval (CI) was calculated. Subgroup analyses according to all the covariates were performed. The age groups were divided into <55 years old and ≥55 years old groups because previous studies demonstrated the effects of estrogen on the occurrence of TC [23]. Thus, it was supposed that the association of psoriasis with TC can be different after menopause in women.

The statistical significance was set as *p* values < 0.05. SAS version 9.4 (SAS Institute Inc., Cary, NC, USA) was utilized.

## 3. Results

A total of 1.35% (92/6822) of the TC group and 1.33% (364/27,288) of the control group documented a history of psoriasis (Table 1). The number of obese patients and current or past smokers as well as SBP, DBP, CCI scores, and histories of hypothyroidism, goiter, thyrotoxicosis, and thyroiditis were higher in the TC group than in the control group. After overlap weighting adjustment, the differences in these variables were diminished. The rates of psoriasis were 1.42% in the TC group and 1.41% in the control group after overlap weighting adjustment.

**Figure 2:**
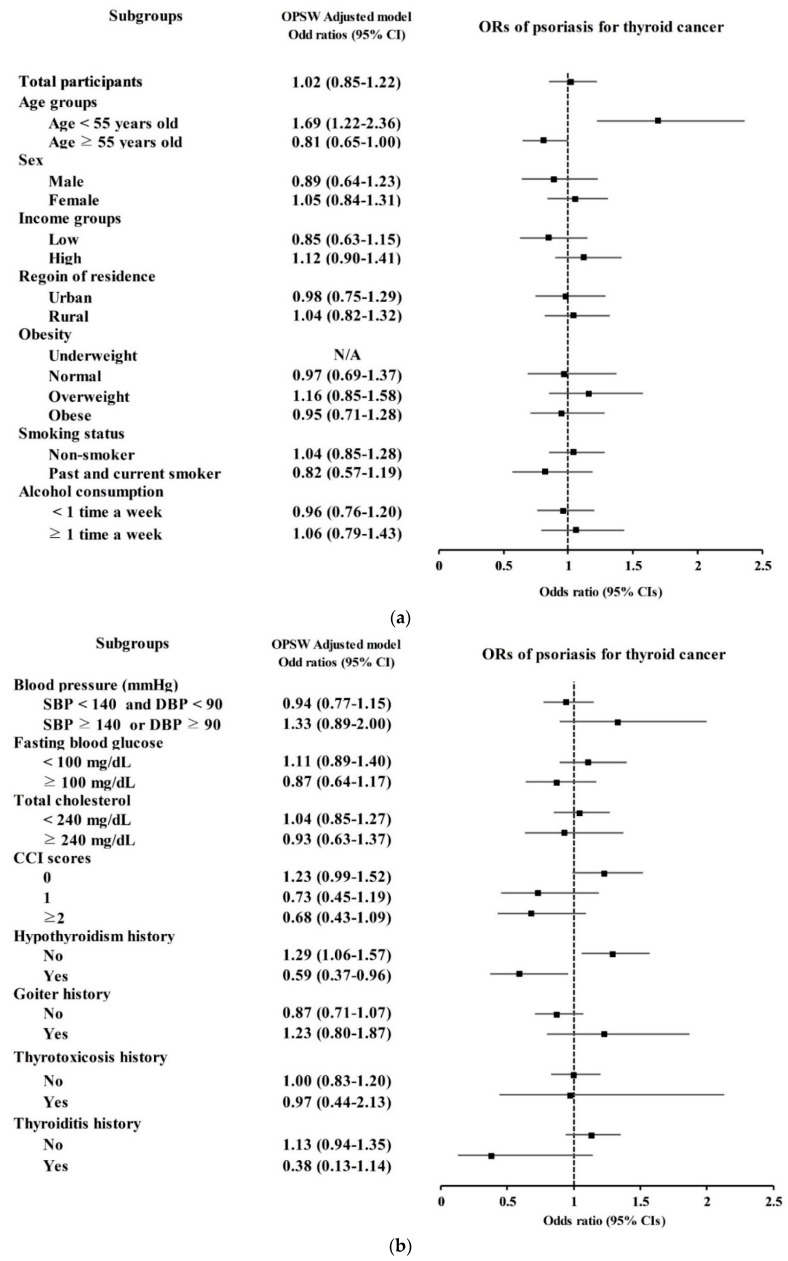
(**a**) Odds ratios of psoriasis for thyroid cancer; (**b**) odds ratios of psoriasis for thyroid cancer according to subgroups.

The TC group was not estimated to have higher odds for psoriasis (overlap-weighted OR = 1.02, 95% CI = 0.85–1.22, *p* = 0.865, Table 2 and Figure 2a). The odds of TC for psoriasis were high in the <55-year-old group (overlap-weighted OR = 1.69, 95% CI = 1.22–2.36, *p* = 0.002). Other subgroups of age, sex, income, and region of residence did not show a significant association between TC and psoriasis.

According to the results regarding the presence of hypothyroidism, the group without hypothyroidism showed high odds of TC for psoriasis (overlap-weighted OR = 1.29, 95% CI = 1.06–1.57, *p* = 0.012, Table 3 and Figure 2b). In contrast, the hypothyroidism group demonstrated low odds of TC for psoriasis (overlap-weighted OR = 0.59, 95% CI = 0.37–0.96, *p* = 0.034). Other subgroups of obesity, smoking status, alcohol consumption, blood pressure, fasting blood glucose, total cholesterol, CCI score, and history of goiter, thyrotoxicosis, and thyroiditis did not report an association between TC and psoriasis.

## 4. Discussion

A history of psoriasis was not associated with TC in the overall adult population. The history of psoriasis was related to TC only in the young adult population (<55 years old) and the population without hypothyroidism. In contrast, patients with histories of hypothyroidism demonstrated a low rate of TC related to a history of psoriasis. The present results clarified the previous studies on the impact of psoriasis on TC.

A few prior studies have suggested an increased risk of TC in patients with psoriasis, with some conflicting results [16]. A case‒control study demonstrated a 1.113 times higher risk of TC in patients with mild psoriasis (95% CI = 1.072–1.156) [16]. However, the risk of TC was not high in patients with severe psoriasis (hazard ratio = 1.127, 95% CI = 0.980–1.297) [16]. In addition, an observational study reported no significant association of psoriasis with TC (adjusted hazard ratio = 1.10, 95% CI = 0.87–1.39) [9]. Another observational study also reported no difference in the standardized incidence ratios of TC related to psoriasis [24]. The current study demonstrated a positive association of psoriasis with TC in specific populations of young adults and patients without hypothyroidism. Thus, it can be presumed that the impact of psoriasis on TC may not be high in the general population.

Immune dysfunction and chronic inflammation in psoriasis patients can be associated with the risk of TC. The activation of T-helper 17 and interleukin-23 cascades has been acknowledged to cause inflammatory skin diseases in patients with psoriasis [25]. These immune dysfunctions, including autoimmunity, have also been regarded as contributors to the occurrence of autoimmune thyroid disease and TC [26,27,28]. In addition, TC, especially well-differentiated papillary thyroid carcinoma, is accompanied by the infiltration of inflammatory cells, such as lymphocytes, macrophages, and mast cells, which promote carcinogenesis [6]. For instance, a case report demonstrated the resolution of psoriasis following the removal of TC [29]. The authors supposed that cytokine release from tumor cells, including tumor necrosis factor-alpha and epidermal growth factor, can induce the maturation of epidermal keratinocytes [29].

In addition to the common immune and inflammatory pathophysiology, a number of comorbidities can mediate the links between psoriasis and TC. For instance, obesity is a risk factor for both psoriasis and TC [4,11]. Thus, obesity can increase the risk of both diseases. As the present study adjusted numerous covariables, the potential mediating effects of these factors may be minimal, in that the association of psoriasis with TC can be attenuated. Moreover, psoriasis-associated risk factors can also increase the risk of cancers, and the treatment of psoriasis, including immunomodulatory drugs, has been suggested to contribute to the risk of overall or site-specific cancers in patients with psoriasis [14]. However, some studies opposed the association of the treatment of psoriasis with biologic agents with the risk of cancer (relative risk = 0.97, 95% CI = 0.85–1.10) [15].

The young adult population described a high rate of history of psoriasis in patients with TC in this study. This can originate from the low prevalence of comorbidities in young adults and the relatively high contribution of psoriasis-associated immune or inflammatory factors in these populations. On the other hand, in the older population, comorbid conditions that may be more common than in the younger population can influence the risk of TC; thus, the impact of psoriasis on the occurrence of TC can be attenuated in this population. Likewise, the participants without a history of hypothyroidism showed a high rate of a history of psoriasis in patients with TC in this study. In contrast, the patients with a history of hypothyroidism documented a low rate of a history of psoriasis.

Hypothyroidism has been presumed to be a risk factor for TC [30]. Thus, the influence of hypothyroidism on the risk of TC mitigates the potential impact of psoriasis on the risk of TC.

This study used a large population cohort in which an unbiased control population can be selected. In addition, overlap weighting adjustment was conducted to attenuate the possible selection bias. Many variables of past medical histories and lifestyle factors were evaluated to minimize possible confounder effects. However, the types and stages of TC were heterogeneous in this cohort. Likewise, the types and severity of psoriasis were also diverse. The medication histories related to thyroid function and psoriasis could not be accounted for. As this study has a retrospective study design, the causal relationship between psoriasis and TC could not be determined. Upcoming studies with prospective study designs can solve the current limitations.

## 5. Conclusions

A history of psoriasis was not related to TC in the adult population. Only the young adult population and adults without hypothyroidism showed a high rate of psoriasis associated with TC.

## Figures and Tables

**Table 1:** General characteristics of participants after and before overlap propensity score weighting.

Characteristics	After Overlap Weighting Adjustment			Before Overlap Weighting Adjustment		
	Thyroid Cancer	Control	Standardized Difference	Thyroid Cancer	Control	Standardized Difference
Age (n, %)			0.00			0.00
40–44	63 (1.79)	63 (1.79)		128 (1.88)	512 (1.88)	
45–49	417 (11.83)	417 (11.83)		837 (12.27)	3348 (12.27)	
50–54	842 (23.86)	842 (23.86)		1623 (23.79)	6492 (23.79)	
55–59	875 (24.80)	875 (24.80)		1672 (24.51)	6688 (24.51)	
60–64	645 (18.26)	645 (18.26)		1194 (17.50)	4776 (17.50)	
65–69	373 (10.57)	373 (10.57)		746 (10.94)	2984 (10.94)	
70–74	218 (6.18)	218 (6.18)		433 (6.35)	1732 (6.35)	
75–79	70 (1.97)	70 (1.97)		144 (2.11)	576 (2.11)	
80–84	25 (0.69)	25 (0.69)		43 (0.63)	172 (0.63	
85+	2 (0.05)	2 (0.05)		2 (0.03)	8 (0.03)	
Sex (n, %)			0.00			0.00
Male	726 (20.57)	726 (20.57)		1513 (22.18)	6052 (22.18)	
Female	2803 (79.43)	2804 (79.43)		5309 (77.82)	21,236 (77.82)	
Income (n, %)			0.00			0.00
1 (lowest)	446 (12.63)	446 (12.63)		852 (12.49)	3408 (12.49)	
2	418 (11.85)	418 (11.85)		803 (11.77)	3212 (11.77)	
3	537 (15.22)	537 (15.22)		1040 (15.24)	4160 (15.24)	
4	745 (21.10)	745 (21.10)		1433 (21.01)	5732 (21.01)	
5 (highest)	1383 (39.20)	1383 (39.20)		2694 (39.49)	10,776 (39.49)	
Region of residence (n, %)			0.00			0.00
Urban	1697 (48.07)	1697 (48.07)		3221 (47.21)	12,884 (47.21)	
Rural	1833 (51.93)	1833 (51.93)		3601 (52.79)	14,404 (52.79)	
Obesity ^†^ (n, %)			0.00			0.13
Underweight	56 (1.58)	56 (1.58)		93 (1.36)	578 (2.12)	
Normal	1212 (34.35)	1212 (34.35)		2210 (32.40)	10,107 (37.04)	
Overweight	984 (27.89)	984 (27.89)		1936 (28.38)	7453 (27.31)	
Obese I	1141 (32.32)	1141 (32.32)		2301 (33.73)	8248 (30.23)	
Obese II	136 (3.86)	136 (3.86)		282 (4.13)	902 (3.31)	
Smoking status (n, %)			0.00			0.08
Nonsmoker	3069 (86.95)	3069 (86.95)		5904 (86.54)	23,182 (84.95)	
Past smoker	252 (7.15)	252 (7.15)		520 (7.62)	1945 (7.13)	
Current smoker	208 (5.90)	208 (5.90)		398 (5.83)	2161 (7.92)	
Alcohol consumption (n, %)			0.00			0.04
<1 time a week	2660 (75.37)	2660 (75.37)		5158 (75.61)	20,176 (73.94)	
≥1 time a week	869 (24.63)	869 (24.63)		1664 (24.39)	7112 (26.06)	
SBP (Mean, SD)	124.14 (11.29)	124.14 (5.74)	0.00	124.59 (15.74)	123.89 (16.17)	0.04
DBP (Mean, SD)	77.04 (7.38)	77.04 (3.76)	0.00	77.41 (10.32)	76.77 (10.48)	0.06
FBG (Mean, SD)	98.14 (16.35)	98.14 (8.51)	0.00	98.00 (23.09)	98.67 (26.09)	0.03
Total cholesterol (Mean, SD)	199.61 (27.40)	199.61 (13.55)	0.00	198.76 (38.05)	201.47 (37.94)	0.07
CCI score (Mean, SD)	0.46 (0.61)	0.46 (0.34)	0.00	0.48 (0.87)	0.42 (0.89)	0.44
Hypothyroidism (n, %)	1233 (34.93)	1233 (34.93)	0.00	3813 (55.89)	2289 (8.39)	1.18
Goiter (n, %)	1375 (38.97)	1375 (38.97)	0.00	3832 (56.17)	3062 (11.22)	1.08
Thyrotoxicosis (n, %)	315 (8.92)	315 (8.92)	0.00	697 (10.22)	1294 (4.74)	0.21
Thyroiditis (n, %)	340 (9.62)	340 (9.62)	0.00	619 (9.07)	1230 (4.51)	0.18
Psoriasis (n, %)			0.01			0.00
No	3479 (98.58)	3480 (98.59)		6730 (98.65)	26,924 (98.67)	
Yes	50 (1.42)	50 (1.41)		92 (1.35)	364 (1.33)	

Abbreviations: CCI, Charlson Comorbidity Index; SBP, systolic blood pressure; DBP, diastolic blood pressure; FBG, fasting blood glucose; PS, propensity score. ^†^ Obesity (BMI, body mass index, kg/m^2^) was categorized as <18.5 (underweight), ≥18.5 to <23 (normal), ≥23 to <25 (overweight), ≥25 to <30 (obese I), and ≥30 (obese II).

**Table 2:** Crude and propensity score overlap-weighted odds ratios of psoriasis for thyroid cancer with subgroup analyses according to age, sex, income, and region of residence.

Characteristics	N of Thyroid Cancer	N of Control	Odd Ratios for Thyroid Cancer (95% Confidence Interval)			
	(Exposure/Total, %)	(Exposure/Total, %)	Crude ^†^	*p* Value	Overlap Weighted Model ^†^	*p* Value
Total participants (n = 34,110)						
Control	6730/6822 (98.7)	26,924/27,288 (98.7)	1		1	
Psoriasis	92/6822 (1.3)	364/27,288 (1.3)	1.01 (0.80–1.27)	0.925	1.02 (0.85–1.22)	0.865
Age < 55 years old (n = 12,940)						
Control	2552/2588 (98.6)	10,250/10,352 (99.0)	1		1	
Psoriasis	36/2588 (1.4)	102/10,352 (1.0)	1.42 (0.97–2.08)	0.073	1.69 (1.22–2.36)	0.002 *
Age ≥ 55 years old (n = 21,170)						
Control	4178/4234 (98.7)	16,674/16,936 (98.5)	1		1	
Psoriasis	56/4234 (1.3)	262/16,936 (1.5)	0.85 (0.64–1.14)	0.284	0.81 (0.65–1.00)	0.054
Male (n = 7565)						
Control	1482/1513 (98.0)	5925/6052 (97.9)	1		1	
Psoriasis	31/1513 (2.0)	127/6052 (2.1)	0.98 (0.66–1.45)	0.905	0.89 (0.64–1.23)	0.486
Female (n = 26,545)						
Control	5248/5309 (98.9)	20,999/21,236 (98.9)	1		1	
Psoriasis	61/5309 (1.1)	237/21,236 (1.1)	1.03 (0.78–1.37)	0.837	1.05 (0.84–1.31)	0.653
Low-income group (n = 13,475)						
Control	2663/2695 (98.8)	10,626/10,780 (98.6)	1		1	
Psoriasis	32/2695 (1.2)	154/10,780 (1.4)	0.83 (0.57–1.22)	0.338	0.85 (0.63–1.15)	0.286
High-income group (n = 20,635)						
Control	4067/4127 (98.5)	16,298/16,508 (98.7)	1		1	
Psoriasis	60/4127 (1.5)	210/16,508 (1.3)	1.14 (0.86–1.53)	0.358	1.12 (0.90–1.41)	0.318
Urban resident (n = 16,105)						
Control	3182/3221 (98.8)	12,717/12,884 (98.7)	1		1	
Psoriasis	39/3221 (1.2)	167/12,884 (1.3)	0.93 (0.66–1.33)	0.705	0.98 (0.75–1.29)	0.892
Rural resident (n = 18,005)						
Control	3548/3601 (98.5)	14,207/14,404 (98.6)	1		1	
Psoriasis	53/3601 (1.5)	197/14,404 (1.4)	1.08 (0.79–1.46)	0.633	1.04 (0.82–1.32)	0.737

Abbreviations: CCI, Charlson Comorbidity Index; GERD, gastroesophageal reflux disease; SBP, systolic blood pressure; DBP, diastolic blood pressure. * Significance at *p* < 0.05; ^†^ adjusted for age, sex, income, region of residence, SBP, DBP, fasting blood glucose, total cholesterol, obesity, smoking, alcohol consumption, CCI scores, hypothyroidism, goiter, thyrotoxicosis, and thyroiditis.

**Table 3:** Subgroup analyses of crude and propensity score overlap-weighted odds ratios of psoriasis for thyroid cancer.

Characteristics	N of Thyroid Cancer	N of Control	Odd Ratios for Thyroid Cancer (95% Confidence Interval)			
	(Exposure/Total, %)	(Exposure/Total, %)	Crude ^†^	*p* Value	Overlap Weighted Model ^†^	*p* Value
Obesity						
Underweight (n = 764)	0/186 (0.0)	8/578 (1.4)	N/A		N/A	
Normal (n = 12,317)	23/2210 (1.0)	112/10,107 (1.1)	0.94 (0.60–1.48)	0.787	0.97 (0.69–1.37)	0.883
Overweight (n = 9389)	33/1936 (1.7)	104/7453 (1.4)	1.23 (0.83–1.82)	0.313	1.16 (0.85–1.58)	0.353
Obese (n = 11,733)	36/2583 (1.4)	140/9150 (1.5)	0.91 (0.63–1.32)	0.615	0.95 (0.71–1.28)	0.745
Smoking status						
Nonsmoker (n = 29,086)	68/5904 (1.2)	270/23,182 (1.2)	0.99 (0.76–1.29)	0.934	1.04 (0.85–1.28)	0.692
Past and current smoker (n = 5024)	24/918 (2.6)	94/4106 (2.3)	1.15 (0.73–1.80)	0.557	0.82 (0.57–1.19)	0.303
Alcohol consumption						
<1 time a week (n = 25,334)	55/5158 (1.1)	241/20,176 (1.2)	0.89 (0.66–1.20)	0.445	0.96 (0.76–1.20)	0.696
≥1 time a week (n = 8776)	37/1664 (2.2)	123/7112 (1.7)	1.29 (0.89–1.87)	0.176	1.06 (0.79–1.43)	0.690
Blood pressure (mmHg)						
SBP < 140 and DBP < 90 (n = 27,315)	70/5455 (1.3)	292/21,860 (1.3)	0.96 (0.74–1.25)	0.764	0.94 (0.77–1.15)	0.56
SBP ≥ 140 or DBP ≥ 90 (n = 6795)	22/1367 (1.6)	72/5428 (1.3)	1.22 (0.75–1.97)	0.424	1.33 (0.89–2.00)	0.166
Fasting blood glucose (mg/dL)						
<100 (n = 22,864)	62/4591 (1.4)	233/18,273 (1.3)	1.06 (0.80–1.41)	0.681	1.11 (0.89–1.40)	0.347
≥100 (n = 11,246)	30/2231 (1.3)	131/9015 (1.5)	0.92 (0.62–1.38)	0.700	0.87 (0.64–1.17)	0.360
Total cholesterol (mg/dL)						
<240 (n = 29,100)	76/5898 (1.3)	311/23,202 (1.3)	0.96 (0.75–1.24)	0.759	1.04 (0.85–1.27)	0.722
≥240 (n = 5010)	16/924 (1.7)	53/4086 (1.3)	1.34 (0.76–2.36)	0.307	0.93 (0.63–1.37)	0.703
CCI scores						
0 (n = 22,996)	56/3760 (1.5)	245/19,236 (1.3)	1.17 (0.87–1.57)	0.288	1.23 (0.99–1.52)	0.063
1 (n = 5413)	14/1234 (1.1)	63/4179 (1.5)	0.75 (0.42–1.34)	0.333	0.73 (0.45–1.19)	0.208
≥2 (n = 5701)	22/1828 (1.2)	56/3873 (1.4)	0.83 (0.51–1.36)	0.463	0.68 (0.43–1.09)	0.111
Hypothyroidism history						
No (n = 28,008)	53/3009 (1.8)	327/24,999 (1.3)	1.35 (1.01–1.81)	0.043 *	1.29 (1.06–1.57)	0.012 *
Yes (n = 6102)	39/3813 (1.0)	37/2289 (1.6)	0.63 (0.40–0.99)	0.045 *	0.59 (0.37–0.96)	0.034 *
Goiter history						
No (n = 27,216)	37/2990 (1.2)	330/24,226 (1.4)	0.91 (0.64–1.28)	0.577	0.87 (0.71–1.07)	0.181
Yes (n = 6894)	55/3832 (1.4)	34/3062 (1.1)	1.30 (0.84–1.99)	0.236	1.23 (0.80–1.87)	0.340
Thyrotoxicosis history						
No (n = 32,119)	84/6125 (1.4)	347/25,994 (1.3)	1.03 (0.81–1.31)	0.822	1.00 (0.83–1.20)	0.994
Yes (n = 1991)	8/697 (1.1)	17/1294 (1.3)	0.87 (0.37–2.03)	0.751	0.97 (0.44–2.13)	0.944
Thyroiditis history						
No (n = 32,261)	86/6203 (1.4)	345/26,058 (1.3)	1.05 (0.83–1.33)	0.696	1.13 (0.94–1.35)	0.205
Yes (n = 1849)	6/619 (1.0)	19/1230 (1.5)	0.62 (0.25–1.57)	0.317	0.38 (0.13–1.14)	0.085

Abbreviations: CCI, Charlson Comorbidity Index; GERD, gastroesophageal reflux disease; SBP, systolic blood pressure; DBP, diastolic blood pressure; * significance at *p* < 0.05; ^†^ adjusted for age, sex, income, region of residence, SBP, DBP, fasting blood glucose, total cholesterol, obesity, smoking, alcohol consumption, CCI scores, hypothyroidism, goiter, thyrotoxicosis, and thyroiditis.

## Data Availability

Restrictions apply to the availability of these data. Data were obtained from the Korean National Health Insurance Sharing Service (NHISS) and are available at https://nhiss.nhis.or.kr (accessed on 6 June 2019) with the permission of the NHISS.
